# Heart involvement in transfusion-dependent beta-thalassemia with conventional echocardiography

**DOI:** 10.22088/cjim.12.3.243

**Published:** 2021-04

**Authors:** Hossein Esfahani, Asadolah Tanasan, Mina Rezanejad, Saadat Torabian

**Affiliations:** 1 Department of Pediatric Oncology, Besat Hospital, Hamadan University of Medical Sciences, Hamadan, Iran; 2 Department of Pediatrics, Besat Hospital, Hamadan University of Medical Sciences, Hamadan, Iran; 3 Department of Epidemiology, Hamadan University of Medical Sciences, Hamadan, Iran

**Keywords:** Beta Thalassemia, Blood Transfusion, Echocardiography, Heart

## Abstract

**Background:**

The most important prognostic factor in transfusion-dependent beta-thalassemia is cardiac involvement which is usually evaluated with echocardiography.

**Methods:**

In this cross-sectional study (April 2011 to April 2012), conventional echocardiography was used to assess myocardial performance and valvular involvement (through transvalvular Doppler study) for right and left heart abnormalities in transfusion-dependent beta-thalassemia.

**Results:**

Among the 60 patients, 43 cases had heart problems, 26 (43.3%) of them had left myocardial dysfunction and 11 (18.3%) of them had right myocardial dysfunction, 3 cases had both RV and LV myocardial dysfunction, (based on LVMPI & RVMPI, respectively). In patients with right myocardial dysfunction, 4 cases had pulmonary hypertension (PH) and 3 had both sided myocardial dysfunction. LVMPI and RVMPI significantly increased in patients with cardiac involvement (p<0.001). Serum ferritin levels in patients with and without cardiac involvement were 2427±1788 ng/ml and 1573±592 ng/ml, respectively (P=0.008). All 4 patients who had PH, had been splenectomized. In splenectomized and non-splenectomized patients, LVMPI was 0.37±0.11 and 0.38±0.1 (P=0.589), RVMPI was 0.3±0.07 and 0.25±0.39 (P=0.004), and TR gradient (TRG) was 28±11.8 mmHg and 19.7±5.2 mmHg (P=0.033), respectively. Mean ferritin level in patients with a history of splenectomy (n=31), was 2525±1968 ng/ml and in patients without the history of splenectomy (n=29) was 1821±947 ng/ml (P=0.082).

**Conclusion:**

In addition to left-sided heart involvement, conventional echocardiography revealed right-sided heart involvement in transfusion-dependent thalassemia patients which did not correlate with serum ferritin level in splenectomized patients.

Thalassemia is a genetic disorder with an inheritance of autosomal co-dominant genes, which generates a heterogeneous group of abnormalities in hemoglobin biosynthesis (1, 2), and chronic hemolytic anemia (3). One of the major complications of thalassemia or its treatment is iron overload, which leads to dysfunction of various organs (4). Cardiac involvement is the most important cause of mortality and morbidity in thalassemia major (5). Secondary cardiac dilatation due to anemia always occurs in young patients who have not been treated. Serious heart problems in these patients, which are due to iron deposition in the myocardium, can lead to cardiomyopathy (5), which is the most important cause of these patients’ mortality (6-9).

Evaluation of specific heart involvements in conventional echocardiography can help to early diagnosis of these problems. Recently, a variety of right myocardial involvement has been reported in transfusion-dependent beta-thalassemia, which requires special attention (10-12). Data reevaluation of our sample patients based on those studies was very interesting for us. 

## Method

**Patients’ collection:** In this cross-sectional study, which was conducted for one year from April 2011 to April 2012, 60 beta-thalassemia patients with transfusion dependency were studied. According to research committee approval number, “89-10-35-16”, patients with thalassemia who were regularly transfused and received chelation, were selected and all demographic information (age, sex, annual blood transfusion, serum ferritin levels) and echocardiographic findings were recorded in the questionnaires. 

**Inclusion and Exclusion criteria: **All patients with transfusion-dependent thalassemia who referred to the thalassemia clinic of Besat Hospital, city of Hamedan, were studied. Exclusion criteria were: non-beta thalassemia patients, non-transfusion dependent beta-thalassemia, an association of congenital heart disease and other congenital malformations.

Patients were evaluated for cardiac status by conventional echocardiography, which was performed with Mylab50 and 60 devices, using a 3-4 MHZ phased array probe. Ejection fraction (EF) was obtained by M-mode echocardiography, in which less than 55% was considered as left ventricular systolic dysfunction. A global diastolic and systolic function index (The Myocardial Performance Index (MPI or Tei index)) was calculated, based on transvalvar doppler echocardiography with the formula: "a-b/b", at which "a" is the time of closure to the opening of the mitral valve for the left side and the time of closure to the opening of tricuspid valve for the right side and “b” is the time of blood ejection from aortic valve for the left side and pulmonary valve for the right side of the heart. LV and RV myocardial dysfunction with MPI greater than 0.45 for the left side of the heart and greater than 0.35 for the right side of the heart were defined. Valve gradients were calculated using continuous doppler of each valve, based on Bernoulli formula (p = 4v^2^) MR, AS, and AI were considered as cardiac involvements in thalassemia patients, while mild TR and PI, which were used for the calculation of pulmonary artery pressure, were not considered as valve lesion. Pathologic MR (mitral valve regurgitation) was considered a holosystolic wave with more than 3 meters/second velocity. Continuous Doppler echocardiography of the tricuspid valve was used to calculate tricuspid regurgitation gradient (TRG). TRG above 35 mmHg and end-diastolic PI above 15 mmHg were considered as pulmonary hypertension (PH) and in case the gradient was greater than half of the aortic systolic pressure, PH was considered severe. Serum ferritin level was measured through the Elisa method by an Elisa reader awareness device manufactured by the USA and a Delaware kit. The information inserted in the checklists and questionnaires was analyzed by SPSS (Version 16) software. Quantitative variables were described as mean +/_; standard deviation and quality variables were described as frequency and percentage. Data were analyzed using t-test, correlation coefficient, x^2^ and a p-value less than 0.05.

## Results

60 beta-thalassemia patients with transfusion dependency were studied, 59 patients had thalassemia major and 1 case had intermediate thalassemia. 33(55%) patients were males and 27(45%) were females. The oldest was 37 years old (male) and the youngest was 3 years (male). The highest age frequency was 19, with 71.6% relative frequency. 39 (65%) patients were taking only deferoxamine (desferal), 20 (33.3%) patients were using deferasirox (Exjade, Osveral) as the main chelator, and only one (1.7%) patient was using deferiprone (L1). The earliest onset of blood transfusion was in the first month of age and the latest blood transfusion was at the age of 9 with an average age of 1.5±1.67 years. The minimum age of onset of chelation therapy was one year, the maximum age was 14, and the average was 4.2±2.53 years. The onset of chelation therapy after blood transfusion was immediate in four patients, with a maximum interval of 9.5 years and a mean of approximately 2.72 years. The mean duration of transfusion therapy before the diagnosis of heart involvement was 18.07±7.8 years, and it was 15.84±6.7 years in patients without cardiac involvement until the present study (P=0.2). The mean duration of chelation therapy until the onset of cardiac involvement was 14.6±9.7 years in patients with heart disease and it was 13.1±4.8 years in patients without heart involvement (P=0.3). The mean serum ferritin level in patients with heart involvement was 2427±1788 ng/ml and in patients without cardiac involvement was 1573±592 ng/ml (P=0.008). The lowest amount of ferritin in patients with heart involvement was 258 ng/ml and the highest value was 11530. A total of 43 patients had abnormal cardiac findings (table 1). The mean total blood transfusion in thalassemia major patients with cardiac involvement, was 179.48±83.3 cc/kg per year and in those without cardiac involvement was 176.61±65 cc/kg per year (P=0.8). The lowest blood transfusion per year was 72.7 cc/kg, the highest amount was 300 cc/kg, and the total mean was 178.92±52 cc/kg.

**Table 1:** Comparison of ferritin, LVMPI, and RVMPI in thalassemia patients with and without cardiac involvement

Groups	With Cardiac involvement (n=43) Mean ± SD	without-cardiac involvement (n=17) Mean ± SD	P-value
Ferritin (ng/dl)	2427±1788	1573±592	P=0.008
LVMPI	0.42±0.09	0.25±0.03	P<0.001
RVMPI	0.3±0.06	0.24±0.02	P<0.001

LVMPI: Left Ventricular Myocardial Performance index, RVMPI: Right Ventricular Myocardial Performance index

**Figure 1:**
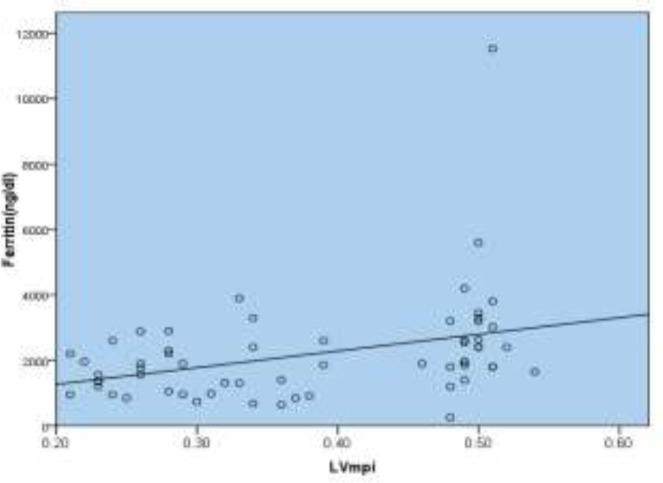
Correlation between Ferritin and LVMPI in the transfusion dependent beta thalassemia:

**Frequency of types of abnormal cardiac findings in thalassemia patients: **43 (71.6%) patients had abnormal cardiac findings according to our definition, including 21 (48.8%) male patients and 22 (51.2%) female patients, in which 26 (43.3%) of them had LV MPI (Tei index) ≥ 0.45, and 11 (18.3%) patients had RVMPI greater than 0.35 (tables 2, 3).

Among these patients, 3 (5%) cases had LVMPI and RVMPI greater than 0.45 and 0.35 respectively. 9 (15%) patients had pathologic MR, which their right and left MPI were within the normal range. Among those with abnormal LVMPI, 9 (15%) patients had E/A ≥1.8, 3 (5%) patients had EF less than 50, 3 (5%) patients had aortic insufficiency, one (1.7%) case had aortic stenosis, and one (1.7%) case had pericardial effusion. Among those with abnormal RVMPI, 3 patients (5%) had mild PH and one (1.7%) patient had severe pulmonary hypertension. Transvalvular continuous Doppler echocardiography studies showed: TR in 29 (46.7%) patients, MR in 18 (30%), AI in 3 (5%) patients and AS in one (1.7%) case. 

**Table 2:** Comparison of Ferritin with LVMPI in the left cardiac dysfunction

Variables	Thalassemia Major without left cardiac dysfunction(N=34)	Thalassemia Major with left cardiac dysfunction (N=26)	P-value
Ferritin (ng/dl)	1684±817	2069±405	0.011
LVMPI	0.29±0.05	0.49±0.1	<0.001

LVMPI: Left Ventricular Myocardial Performance index

**Table 3:** Comparison of Ferritin with RVMPI in the right cardiac dysfunction

Variables	Thalassemia Major without right cardiac dysfunction (N=49)	Thalassemia Major with right cardiac dysfunction (N=11)	P-Value
Ferritin (ng/dl)	1839±867	3726±2840	0.053
RVMPI	0.27±0.04	0.43±0.01	<0.001

RVMPI: Right Ventricular Myocardial Performance index

Pearson correlation showed a correlation between ferritin level and LVMPI with P=0.005 (figure1) and correlation between ferritin and RVMPI with P<0.001 but there was no relationship between ferritin levels and TR gradients (P=0.1). Splenectomy was done in 31 patients with thalassemia, among them 22 patients had the cardiac disease (71%). On the other hand, among the 43 patients with cardiac disease, 22 (51.2%) patients had been splenectomized (P=0.9). Of the 26 patients with left myocardial dysfunction, 12 (46.1%) patients had been splenectomized, and of the 31 patients with a history of splenectomy, 12(38.7%) had left and 10 (32.2%) patients had right myocardial dysfunction respectively. Among 11 patients with right ventricular dysfunction, 10 (90.9%) patients had history of splenectomy (P=0.004). Splenectomy was done in all 4 cases with elevated pulmonary artery pressure. Among splenectomized patients, tricuspid regurgitation (TR) was seen in 18 (58.1%) patients, and 13 cases did not develop TR (41.9%)) which statistically is not significant (P=0.22) but the comparison of TRG between two groups was significant (table 4). Splenectomy was done at the mean age of 9.45±5.9 years in patients with heart disease, and 8.27±3.5 years in normal heart subjects (P=0.5).

**Table 4:** Comparison of ferritin, LVMPI, RVMPI, and TRG between patients with and without history of splenectomy

**Variables**	P-Value	With Splenectomy history (n=31)	Without Splenectomy history (n=29)	P-Value
Ferritin (ng/dl)	0.082	2525±1968	1821±947	0.082
LVMPI	0.589	0.37±0.11	0.38±0.1	0.589
RVMPI	0.004	0.3±0.07	0.25±0.39	0.004
TRG (mmHg)	0.033	28±11.8	19.7±5.2	0.033

LVMPI: Left Ventricular Myocardial Performance index, RVMPI: Right Ventricular Myocardial Performance index, TRG: Tricuspid Regurgitation Gradient

## Discussion

Chronic anemia in thalassemia major (1) has a profound effect on various organs (3, 4), especially in the heart, which is the main cause of mortality and morbidity in these patients (5). In the early stages, heart involvement is sub-clinical and is shown as diastolic dysfunction, which can be diagnosed during echocardiography (7). The importance of evaluation at this stage is due to the likelihood of progression to more severe cardiac problems, which may present as systolic dysfunction. In advanced stages of the disease, which cause cardiac systolic dysfunction, and other life-threatening heart complications are also more likely to develop (8).

In this study, which is conducted on 60 patients with transfusion-dependent beta-thalassemia using conventional echocardiography, left heart myocardial dysfunction was seen in 43.3% of patients, based on LVMPI (as a global diastolic and systolic function index), while only 5% of patients had left ventricular ejection fraction abnormality, based on LVEF (as an LV systolic function index). 9 patients (15%) had a mean mitral E/A ratio greater 1.8, which may be seen in diastolic myocardial dysfunction. This finding may express that MPI in this patients was more valuable in diastolic dysfunction than systolic dysfunction. Besides, this study shows that the myocardial performance index in conventional echocardiography was significantly disturbed in the right side of the heart as well as the left side and can be used as a good marker for right heart myocardial dysfunction. Among the 11cases with right myocardial dysfunction, eight patients had isolated right-sided heart problems, 4 of whom were associated with increased pulmonary artery pressure (6.6%), which was close to the percentage obtained in another study in Iran (4.5%) (10). As there are some other studies, which report the right heart involvement and pulmonary hypertension in asymptomatic patients (11), consideration of those issues in the follow up is important. While some studies suggest the relationship of increased pulmonary artery pressure with high serum ferritin level and splenectomy (12, 13) other studies showed a low risk of pulmonary hypertension in well-transfused beta-thalassemia patients (13). In the present study, there was a significant relationship between splenectomy and right heart involvement, especially pulmonary hypertension that specific treatments may help these patients (14). Interestingly in our study, 17 (28.3%) patients had a cardiac involvement with normal LVMPI. Mitral and aortic valve involvements have been reported in 30% and 5%, respectively compared to a multicenter study on thalassemia intermedia among whom mitral valve involvements were seen in 42.7% and aortic valve involvements were seen in 15.4% of their cases (15). In our study, 15% of patients with MR had MR without myocardial dysfunction and the rest had left myocardial dysfunction, which many interpret as valve lesions progression independent of cardiac function in these patients, that sometimes requires intensive treatment (16-17). 

In the present study, there was a clear relationship between the level of ferritin and cardiac involvement, as reported in many other studies. It means that overall the higher serum ferritin levels are associated with a higher risk of cardiac damage. In the present study, there was no significant difference between the level of ferritin and the total amount of blood transfusion per year in patients, which may be due to other mechanisms that increase our patients’ ferritin levels (15) or it may be due to effective iron chelation protocols (16) or even differences in iron metabolism in each person (17). In our study, 43.3% of patients had LV dysfunction and 18.3% had RV dysfunction and the relationship between ferritin and LVMPI and RVMPI were significant. In comparison between patients with and without cardiac involvement, there was a significant correlation between serum ferritin level and LV dysfunction based on LVMPI in the conventional echocardiography (P=0.011) that this correlation was showed by advanced echocardiography in tissue Doppler imaging indexes (6). In the present study, the correlation between serum ferritin and RV dysfunction was not significant (P=0.053) in which further analysis revealed that it is correlated to splenectomized patient group. In our study, there were 31 patients with a history of splenectomy, 22 (71%) of whom had heart disease. Serum ferritin levels of these patients were higher than non-splenectomized patients (table 4), although this difference was not significant (P=0.082).

According to LVMPI (global criteria for systolic and diastolic performance), 38.7% of splenectomized patients had left myocardial dysfunction, which was not significant in comparison with non-splenectomized cases. In a recent study, researchers used mitral valve filling parameters, left atrial volume index based on cardiac MRI (CMR), left ventricular diastolic dysfunction in patients with splenectomy was evident (18). 

Increased iron deposition in the heart, based on the T2 CMR reports in patients with the history of splenectomy has been reported in some articles (19). In another study, ferritin level, tricuspid regurgitation velocity (TRV) and diastolic dysfunction of the right ventricle were higher in patients with a history of splenectomy (12) which is consistent with our study in which the right ventricular dysfunction and TRG (TR gradient) were higher in patients with a history of splenectomy. However in our study, there was no significant relationship between TR occurrence and history of splenectomy (P=0.22) and also TRG was not correlated with serum ferritin level (P=0.1) that is not consistent with some studies (12). It can be concluded that in our patients and especially in patients with splenectomy, right ventricular dysfunction and increased pulmonary artery pressure probably have other causes than increased serum ferritin levels (13, 20). 

In the present study, although 32.2% of patients with splenectomy had right cardiac dysfunction based on RVMPI criterion, approximately 91% of patients with right ventricular dysfunction had a history of splenectomy (P=0.004) and all four patients with increased pulmonary artery pressure (PH) had been splenectomized, and given that left ventricular dysfunction and high serum ferritin elevation in these patients were not clear, other mechanisms have been suggested for the cause of PH in these patients (21). Platelet activation and hypercoagulability state are among the proposed considering factors in the association of PH and splenectomy (22). This hypothesis can be considered for investigations in future research. 

In addition to left-sided heart involvement, conventional echocardiography revealed right-sided heart involvement in transfusion-dependent thalassemia patients. While in patients with history of splenectomy, right myocardial dysfunction and increased pulmonary artery pressure without correlation with serum ferritin level can be seen.

## Competing:

No financial or nonfinancial benefits have been received or will be received from any party related directly or indirectly to the subject of this article

## References

[1] Galanello R, Origa R (2010). Beta-thalassemia. Orphanet J Rare Dis.

[2] Kasper D, Fauci A, Hauser S (2015). Harrison's principles of internal medicine. 19th ed. New York: McGraw-Hill Education.

[3] Mehmood R, Yaqoob U, Sarfaraz A, Zubair U (2018). Complete blood picture with skeletal and visceral changes in patients with thalassemia major. Int J Health Sci (Qassim).

[4] Cunningham MJ, Macklin EA, Neufeld EJ, Cohen AR; Thalassemia Clinical Research Network (2004). Complications of beta-thalassemia major in North America. Blood.

[5] Li CK, Luk CW, Ling SC (2002). Morbidity and mortality patterns of thalassaemia major patients in Hong Kong: retrospective study. Hong Kong Med J.

[6] Nanjegowda CK, Kamath SP, Kamath P (2019). Comparison of diastolic function in children with transfusion dependent beta thalassemia major by tissue and conventional doppler imaging indices and its correlation with serum ferritin levels. Turk J Pediatr.

[7] Gharzuddine WS, Kazma HK, Nuwayhid IA (2002). Doppler characterization of left ventricular diastolic function in beta-thalassaemia major. Evidence for an early stage of impaired relaxation. Eur J Echocardiogr.

[8] Hamed AA, Elguindy W, Elhenawy YI, Ibrahim RH (2016). Early cardiac involvement and risk factors for the development of arrhythmia in patients with beta-thalassemia major. J Pediatr Hematol Oncol.

[9] Mancuso L, Vitrano A, Mancuso A (2018). Left Ventricular diastolic dysfunction in β-thalassemia major with heart failure. Hemoglobin.

[10] Rashidi F, Sate H, Mohammadi A (2018). Echocardiographic evaluation of prevalence of pulmonary hypertension in beta-thalassemia major: A cross sectional study. Pediatr Hematol Oncol.

[11] Noori NM, Keshavarz K, Shahriar M (2012). Cardiac and pulmonary dysfunction in asymptomatic beta-thalassanemia major. Asian Cardiovasc Thorac Ann.

[12] Dedeoglu S, Bornaun H (2017). Pulmonary hypertension in children with beta thalassemia major, are splenectomy and high-ferritin levels related or not?. J Pediatr Hematol Oncol.

[13] Meloni A, Detterich J, Pepe A (2015). Pulmonary hypertension in well-transfused thalassemia major patients. Blood Cells Mol Dis.

[14] Karami H, Darvishi-Khezri H, Kosaryan M, Akbarzadeh R, Dabirian M (2019). The improvement of pulmonary artery pressure after bosentan therapy in patients with beta-thalassemia and Doppler-defined pulmonary arterial hypertension. Int Med Case Rep J.

[15] Koohi F, Kazemi T, Miri-Moghaddam E (2019). Cardiac complications and iron overload in beta thalassemia major patients-a systematic review and meta-analysis. Ann Hematol.

[16] Kolnagou A, Kontoghiorghe CN, Kontoghiorghes GJ (2017). Prevention of iron overload and long term maintenance of normal iron stores in thalassaemia major patients using deferiprone or deferiprone deferoxamine combination. Drug Res (Stuttg).

[17] Zhang H, Zhabyeyev P, Wang S, Oudit GY (2019). Role of iron metabolism in heart failure: From iron deficiency to iron overload. Biochim Biophys Acta Mol Basis Dis.

[18] Chinprateep B, Ratanasit N, Kaolawanich Y (2019). Prevalence of left ventricular diastolic dysfunction by cardiac magnetic resonance imaging in thalassemia major patients with normal left ventricular systolic function. BMC Cardiovasc Disord.

[19] Aydinok Y, Bayraktaroglu S, Yildiz D, Alper H (2011). Myocardial iron loading in patients with thalassemia major in Turkey and the potential role of splenectomy in myocardial siderosis. J Pediatr Hematol Oncol.

[20] Fayed MA, Abdel-Hady HE, Hafez MM, Salama OS, Al-Tonbary YA (2018). Study of platelet activation, hypercoagulable state, and the association with pulmonary hypertension in children with beta-thalassemia. Hematol Oncol Stem Cell Ther.

[21] Anthi A, Orfanos SE, Armaganidis A (2013). Pulmonary hypertension in beta thalassaemia. Lancet Respir Med.

[22] Manakeng K, Prasertphol P, Phongpao K (2019). Elevated levels of platelet- and red cell-derived extracellular vesicles in transfusion-dependent beta-thalassemia/HbE patients with pulmonary arterial hypertension. Ann Hematol.

